# Identification, Characterization, and Antioxidant Potential of *Bifidobacterium longum* subsp. *longum* Strains Isolated From Feces of Healthy Infants

**DOI:** 10.3389/fmicb.2021.756519

**Published:** 2021-11-02

**Authors:** Li Zhao, Song Wang, Jiahuan Dong, Jialu Shi, Jiaqi Guan, Deyu Liu, Fei Liu, Bailiang Li, Guicheng Huo

**Affiliations:** ^1^Key Laboratory of Dairy Science, Ministry of Education, Northeast Agricultural University, Harbin, China; ^2^College of Food Science, Northeast Agricultural University, Harbin, China

**Keywords:** *Bifidobacterium longum* subsp. *longum* strain, infant, potential probiotics, antibiotics susceptibility, cell proliferation, antioxidant

## Abstract

Increasing evidence has indicated that oxidative stress is associated with the health of infants. *Bifidobacterium*, especially *B. longum* subsp. *longum* strains, are abundant in the gut microbiota of infants, which may have the potential to ameliorate oxidative damage. Thus, this study aimed to isolate and screen *B. longum* subsp. *longum* strains with probiotic characters and antioxidant properties as infants’ dietary supplements. In this study, 24 *B. longum* subsp. *longum* strains were isolated from 15 healthy infants identified *via* 16S rRNA and heat shock protein 60 (*hsp*60) sequences. *B. longum* subsp. *longum* B13, F2, K4, K5, K10, K13, and K15 strains were selected based on high values obtained from autoaggregation, hydrophobicity, and adhesion assays to HT-29 cells. Among these seven strains, *B. longum* subsp. *longum* F2, K5, K10, and K15 were selected according to the high tolerance of gastrointestinal tract conditions compared to *Bifidobacterium animalis* subsp. *lactis* BB-12. Among these four strains, *B. longum* subsp. *longum* K5 was susceptible to common antibiotics and showed the highest intestinal epithelial cell proliferation of CCD 841 CoN. Additionally, *B. longum* subsp. *longum* K5 showed a strong antioxidant capacity, and its supernatant exhibited better activity of reducing power, hydroxyl radical scavenging, and DPPH radical scavenging than that of the intact cells with cell-free extracts. The findings indicated that *B. longum* subsp. *longum* K5 could be used as a probiotic candidate in infant nutrition.

## Introduction

Oxidative stress has been closely linked to the health of infants, especially preterm infants (Cong et al., 2016). Preterm infants are vulnerable to oxidative damage because of the absence of antioxidants at birth and the impairment of the synthesis ability of antioxidants (Hanson et al., 2016). The undeveloped antioxidant defense system of infants may increase the risk for complications of intestinal and systemic anomalies, such as retinopathy of prematurity, necrotizing enterocolitis, and bronchopulmonary dysplasia (Weber et al., 2014). Furthermore, it has been reported that the level of oxidative stress of preterm infants was higher than that of full-term infants (Cai et al., 2019). Furthermore, dietary antioxidants were related to reducing the risk of inflammation, diabetes, carcinogenesis, and other health problems associated with aging (Uttara et al., 2009). Thus, it is critical to find natural antioxidants which could apply in baby dietary supplements.

*Bifidobacterium* is one of the predominant genera of the infant gastrointestinal tract (Milani et al., 2015; Duranti et al., 2017; Nuriel-Ohayon et al., 2016; Ma et al., 2020). As probiotics, they inhibit pathogenic bacteria colonization and regulate immune response (Ruiz et al., 2017). Thus, Bifidobacteria were usually applied in dietary supplements as probiotics. Furthermore, it has been reported that Bifidobacteria possess excellent antioxidant properties (Mishra et al., 2015). However, most of the Bifidobacteria applied in baby dietary antioxidant supplements are not originated from infants. Before they reach the mature adult gut microbiota, the development proceedings of infant gut microbiota are influenced by many external environmental factors (e.g., antibiotics, food). The infant gut microbiota is inherent and less affected by the environment than the adult gut microbiota. Furthermore, the amount of *Bifidobacterium* strains decreases with age (Silvia et al., 2016), so the current research aims to isolate *Bifidobacterium* from infants. Additionally, evidence has been reported that the abundance and species of *B. longum* colonized in the intestine are closely associated with the health of the body (Akay et al., 2014; Zhang et al., 2019). Thus, it is still a challenge for us to discover potential Bifidobacteria, especially *B. longum*, with antioxidant properties derived from infants.

Being alive and providing beneficial effects in sufficient amounts were the requirements for the probiotic selection claimed by the World Health Organization (Morelli and Capurso, 2012; Nazhand et al., 2020). To be effective, the probiotic should possess the potential ability to adhesion to the intestinal epithelial cells and survive under the gastrointestinal tract (Vankerckhoven et al., 2008; Mandal et al., 2016). The adhesion ability may contribute to the bacteria against undesirable bacteria by competition for binding sites of host intestinal epithelial cells and increasing the chances to interrelate with the host (Celebioglu et al., 2017; Okochi et al., 2017; Monteagudo-Mera et al., 2019). Probiotics demonstrated that the antibiotic resistance genes they carried could be transferred to intestinal pathogens in the host gut (Salyers et al., 2004; Ammor et al., 2007; Sommer et al., 2009). Therefore, it is vital to evaluate the antibiotic resistance properties of bacteria. Furthermore, the development of the intestinal tract is significant in infants; immature intestines and slow intestinal immune development were associated with the high risk of postpartum growth restriction, lactose intolerance, allergy, necrotizing enterocolitis, and other intestinal related diseases (Zhuang et al., 2019; Osorio, 2020; Crovetto et al., 2021). Studies confirmed that probiotics were beneficial to intestinal development, intestinal epithelial renewal, and intestinal mucosa balance (Blumberg and Gensollen, 2017; Zhuang et al., 2019). Thus, it is vital to find effective probiotics to promote intestinal development with no side effects.

The objective of this study was to evaluate and screen *B. longum* subsp. *longum* isolated from infant feces and assess its adhesion ability, high tolerance during gastrointestinal digestion, antibiotic susceptibility, capacity for intestinal epithelial cell proliferation, and antioxidant, which could apply in infants’ dietary antioxidant supplements.

## Materials and Methods

### Bacteria Strains and Cells Culture

Isolates were anaerobically incubated in de Man, Rogosa and Sharpe broth supplemented with 0.05% L-cysteine hydrochloride (MRS_L_) (2% v/v) at 37°C for 24 h and subcultured twice before further experiments. *Bifidobacterium animal* subsp. *Lactis* BB-12 (BB-12) were obtained from the American Type Culture Collection (ATCC).

The normal human colonic epithelial cells CCD 841 CoN cells were kindly donated by Prof. Meng Xiangchen and stored in liquid nitrogen before use. The human colon cell line HT-29 cells were obtained from the Chinese Academy of Sciences (Shanghai, China). Cells were cultured in DMEM (HyClone, United States) supplemented with 10% fetal bovine serum (FBS, AlphaBio, South America) (DMEM_F_) at 37°C in a 5% CO_2_ atmosphere.

### Sample Collection

Fifteen fresh feces samples of healthy infants (aged 0–6 months) without taking antibiotics before the time of sampling were collected from Harbin, Heilongjiang Province of China. The samples were collected in sterile feces collectors and kept at 4°C until transported to the laboratory. All the operations were completed within 2 h for the isolation of Bifidobacteria.

### Isolation and Screening of Bifidobacteria

The samples were diluted and spread on MRS agar with 0.05% L-cysteine (MRS_L_) and incubated at 37°C for 72 h under an anaerobic atmosphere (10% H_2_, 10% CO_2_, and 80% N_2_) (Bevilacqua et al., 2003). Gram-positive colonies were purified on MRS_L_ and incubated under the same conditions. Purified colonies only grown under anaerobic conditions were selected for the assay of catalase activity. Catalase-negative colonies were inoculated in MRS_L_ liquid medium at 37°C for 24 h.

### Identification of Bifidobacteria

#### Identification of Bifidobacteria by 16S rRNA Gene Sequencing

The total DNA was extracted from a 1 mL culture medium of different isolated strains (Liu et al., 2015). The genomic DNA primer is shown in Table 1. The results were sequenced by KuMei Company, Harbin, China, and screened using the BLAST program. The sequence of strains was uploaded in the GenBank (Supplementary Table 1). A phylogenetic was constructed *via* the neighbor-joining method using the software of Mega 6.0.

**TABLE 1:** Primer sequences for amplification.

Gene	Primer sequence (5′→3′)
16S rDNA gene	27 F: 5′-AGAGTTTGATCCTGGCTCAG-3′
	1495 R: 5′-CTACGGCTACCTTGTTACGA-3′
*hsp60* gene	hspF3: 5′-ATCGCCAAGGAGATCGAGCT-3′
	hspR4: 5′-AAGGTGCCGCGGATCTTGTT-3′

#### Identification of Bifidobacteria by Heat Shock Protein 60 Gene

All Bifidobacteria strains identified by 16S rRNA gene sequencing were discriminated *via* the *hsp60* gene (Modesto et al., 2018). The genomic DNA is shown in Table 1. PCR amplification was set in 50 μL volume, including master mix (Yesen, Shanghai, China), 2 μM primer, 120 ng chromosomal DNA, and ddH_2_O. The cycling program was performed at 95°C for 5 min, 35 cycles of 94°C, 30 s, 59°C, 60 s, 72°C for 30 s, and the final extension was set at 72°C for 10 min. A phylogenetic was constructed as described above.

#### Morphology, Scanning Electron Microscopy Analysis

The SEM analysis was determined using a modified method as previously described by Guang-Yu et al. (2017). After incubation, the Bifidobacteria were centrifuged at 3,000 × *g* for 5 min. The pellets were washed three times using phosphate-buffered saline (PBS) solution, mixed with 2.5% glutaraldehyde (pH 6.8), and fixed for at least 1.5 h at 4°C. Samples were obtained by washing three times with PBS after centrifuged, then dehydrated with ethanol. Then the samples were freeze-dried and sputter-coated with 100 Å of gold after being replaced with tert-butanol. The results were obtained with Hitachi S-3400N (Hitachi, Japan) SEM.

### Adhesion Properties

#### Autoaggregation

Autoaggregation assay was performed according to the method of Krausova et al. (2019). with some modifications Bifidobacteria grown overnight were centrifuged (6,000 × *g*, 10 min) and adjusted to an optical density of 0.6 at 600 nm (OD 600, A_0_). 8 ml of each stain was incubated at 37°C for 1 h, 4 h, and 24 h (A_t_) and then the upper suspension was determined at 600 nm. The autoaggregation was calculated with the following formula:


A(%)=(A-0A)T/A×0100

#### Cell Surface Hydrophobicity

Hydrophobicity assay was performed with the modified method as previously described by Yan et al. (2020). CSH was determined with ethyl-acetate (basic polar solvent) and xylene (apolar solvent). Bifidobacteria were performed as described above. The cell pellets were suspended in PBS to a concentration of 0.8–1.0 at 600 nm (H_0_). Then, 1 mL ethyl-acetate or xylene was mixed with 3 mL cell suspension and shocking for 2 min. The aqueous phase was collected and measured at 600 nm (H_1_) after incubating at 37°C for 1 h. The value of hydrophobicity was calculated as follows:


H(%)=(H-0H)1/H×0100

#### Adhesion of Bifidobacteria to HT-29 Cells

HT-29 cells were incubated at 37°C in a 5% CO2 atmosphere using a pre-heated DMEM_F_ medium. The adhesion assay was determined by the method of Li et al. (2014) with some modifications. HT-29 cells (3.0 × 10^5^ cells/mL) were seeded in 12-well culture plates and changed the medium every 2 days until monolayers of HT-29 cells were confirmed. Then, 1 mL of strains with a 1.0 × 10^8^ cfu/mL concentration was added per well and incubated at 37°C for 2 h. Each well was washed five times with PBS and added 0.25 mL trypsin-EDTA for 10 min to digest. The reaction was terminated *via* adding the same volume of FBS, and the samples of each well were collected and plated on MRS_L_ agar. The results were assessed as follows:


Adhesion(%)=(thenumberofadheredbacteria/1.0×10)8×100%

#### Principal Component Analysis

Principal component analysis (PCA) reduces the dimensionality of the original dataset to a new set of non-correlated variables, called principal components (PCs), without losing much information. Principal component analysis was performed with SPSS 16.0 software on a data matrix containing autoaggregation at 1, 4, and 24 h, CSH in ethyl-acetate and xylene, and adhesion of Bifidobacteria to HT-29 cells of different *B. longum* subsp. *longum* strains. The total scores of strains were obtained according to the contribution of components and factor score and used to selected Bifidobacteria with good adhesion ability.

### Simulation of Gastrointestinal Digestion *in vitro*

The survival rate of test strains in the simulated gastrointestinal tract was accessed by the modified method of the previous assay (Zhu et al., 2015). Strains were adjusted to 1.0 × 10^9^ cfu/mL after incubated for 24 h. The cells were added into gastric juice for 2 h, 1 mL of the solution was taken into the intestinal fluid for 2 h incubation. The survival cells were valued by plate counting as the following formula:


$$\mathrm{Survival}\mathrm{rates}\left(\%\right)=\frac{{\text{LgN}}_{1}}{{\text{LgN}}_{0}}\times 100$$

Here, the survival strains were represented by N_1_, and N_0_ stands for the total strains before the treatment.

### Antibiotic Susceptibility

The antibiotic susceptibility of test strains was evaluated against antibiotics vancomycin, gentamicin, penicillin G, chloramphenicol, kanamycin, streptomycin, rifampicin, amoxicillin, tetracycline, erythromycin, roxithromycin, clindamycin, trimethoprim (MTP), ciprofloxacin, and ampicillin, using the disk diffusion method reported by Rajoka et al. (2017). After incubation for 48 h at 37°C, the inhibition zone diameter was measured and analyzed according to the commercial introduction provided by Hangzhou Binhe Microorganism Reagent Co., Ltd., China.

### Cell Proliferation

Cell proliferation of test strains was evaluated by the CCK-8 (Dalian Meilun Biotechnology Co., Ltd., China) method described by Liang et al. (2017). The CCD 841 CoN cells were seeded in 96-well plates and incubated for 24 h to ensure cell attachment. Then, Bifidobacteria strains (MOI 1:100) were added into the cells for 24 h incubation. The medium was discarded, and 100 μL 10% CCK-8 solution was added to each well. After 2 h incubation, each well’s optical density (OD) was detected at 450 nm using a microplate reader (Molecular Devices, United States). Cell proliferation of each sample was calculated with the following equation:


$$\mathrm{Cell}\mathrm{relative}\mathrm{proliferation}\left(\%\right)=\left({\text{OD}}_{\text{sample}}-{\text{OD}}_{\text{blank}}\right)\times 100$$

### Antioxidant Activity

#### Preparation of Samples

The test strains were incubated as above, and the cell-free supernatant (CFS) was collected by centrifugation (8,000 × *g*, 15 min) at 4°C. The intact cells (IC) were harvested by washing three times with PBS (pH 7.4) and then resuspended at a 1.0 × 10^9^ cfu/mL concentration. The cell-free extracts (CFE) were acquired by ultrasonic in an ice bath for 15 min and centrifuged (8,000 × *g*, 15 min) at 4°C for the supernatant. The CFE and CFS were treated with 0.22 mm filter membranes for further experiments.

#### Reducing Power

Reducing activity was determined by the method of Oyaizu (1988). A total of 0.5 mL of PBS (pH 6.6) and potassium ferricyanide (1%) were added into 0.5 mL of each sample. The mixture was cultured at 50°C for 20 min and then cooled to room temperature rapidly. Then, 0.5 mL of trichloroacetic acid (10%) was added, and 1 mL of the supernatant was added into 1.0 mL of ferric chloride (0.1%) after centrifuging (3,000 × *g*, 5 min). The absorbance was recorded at 700 nm after the mixture was placed at room temperature for 10 min. The standard expression of L-Cysteine assessed the value of reducing activity.

#### Hydroxyl Radical Scavenging

A previous study described by Makoto and Oyaizu (1988) analyzed the Hydroxyl radical scavenging assay. A total of 1.0 mL of each sample was added into the mixture contained 2.5 mM 1,10-phenanthroline (1.0 mL), 2.5 mM FeSO_4_ (1.0 mL), and PBS (1.0 mL, pH 7.4). The mixture was incubated in a 37°C water bath for 90 min after adding 20 mM H_2_O_2_ (1.0 mL). The absorbance was obtained at 517 nm. The Hydroxyl radical scavenging activity was calculated as follows:


$$\mathrm{Scavenging}\mathrm{ability}\left(\%\right)=\left(\frac{{\text{A}}_{\text{sample}}-A_{\text{blank}}}{{\text{A}}_{\text{control}}-A_{\text{blank}}}\right)\times 100$$

Here, A_sample_ was represented the absorbance of each test sample, the controls contained distilled water instead of H_2_O_2_ in the reaction system, and the blanks included distilled water instead of samples in the reaction system.

#### DPPH Radical Scavenging

The DPPH radical scavenging activity was measured using the method with some modifications of Lin and Yen (1999). A total of 1.0 mL of each sample was mixed with 1.0 mL of 0.2 mM DPPH radical solution and placed for 30 min in the dark. Then, the mixture was centrifuged at 3,000 × *g* for 10 min. The absorbance was read at 517 nm. The scavenging ability of DPPH radical was assessed as follows:


$$\mathrm{Scavenging}\mathrm{abilit}y\left(\%\right)=\left(1-\frac{{\text{A}}_{\text{sample}}-A_{\text{blank}}}{{\text{A}}_{\text{control}}}\right)\times 100$$

Where *A*_*sample*_ stands for the absorbance of each sample, the controls contain DPPH solution and distilled water, and the blanks include only the samples and ethanol.

#### Superoxide Anion Radical Scavenging Activity

According to a previous assay, the superoxide anion radical scavenging properties were assessed (Li et al., 2012). A total of 3.0 mL of Tris–HCl solution (pH 8.2) was mixed with 1.0 mL of samples and stood at room temperature for 20 min. The mixture was placed in the dark for 4 min after adding 0.4 mL of pyrogallol (25 mM). Then, 0.5 mL of HCL (8 mM) was used to end the reaction. The absorbance was recorded at 325 nm. The ability of superoxide anion radical scavenging was valued as follows:


$$\mathrm{Scavenging}\mathrm{ability}\left(\%\right)=\left(1-\frac{{\text{A}}_{\text{sample}}}{{\text{A}}_{\text{blank}}}\right)\times 100$$

Where A_*sample*_ stands for the absorbance of each sample, and the blanks include distilled water, not samples in the reaction system.

### Statistical Analyses

All tests were executed in triplicate, and the data were expressed as the mean ± standard deviation. Analysis of variance (One-Way ANOVA) was realized with the method of Duncan’s multiple ranges (*p* < 0.05) *via* SPSS statistical software version 16.0 to value the statistical significance of differences between means.

## Results

### Morphology Analysis

**FIGURE 1:**
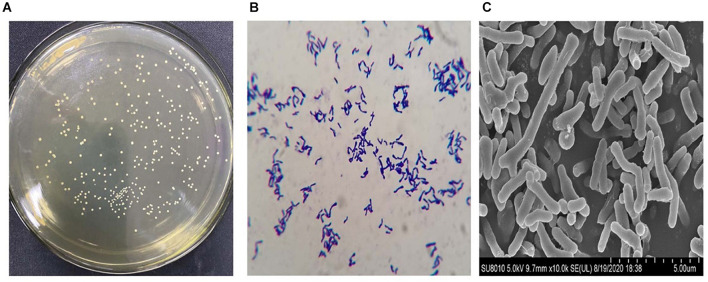
Representative morphologic characteristics of *B. longum* subsp. *longum* strains. **(A)** Colony morphology; **(B)** Gram staining morphology; **(C)** Scanning electron microscopy. Morphology of the *B. longum* subsp. *longum* strain was obtained using a scanning electron microscope ×10.0 K.

The *B. longum* subsp. *longum* strains were generated circular, smooth surface, convex, and white colonies on MRS_*L*_ (Figure 1A). Pleomorphism (dumbbell, V, and Y shaped) was found in the Gram staining of *B. longum* subsp. *longum* (Figure 1B). The morphology of *B. longum* subsp. *longum* was shown in Figure 1C *via* scanning electron microscopy.

### Isolation and Identification of Bifidobacteria

**FIGURE 2:**
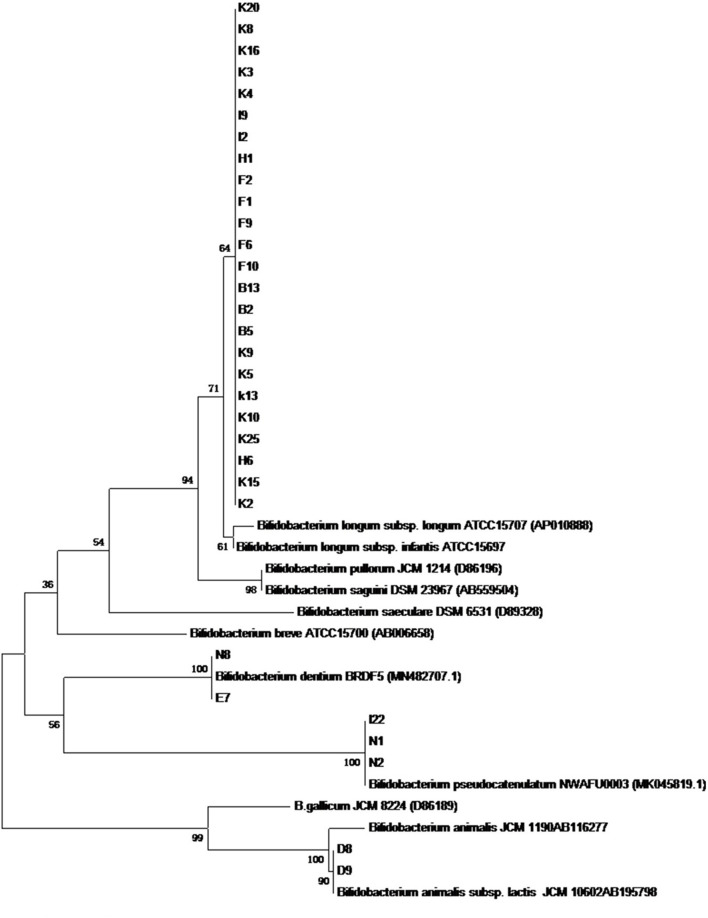
The phylogenetic tree based on 16S rRNA genes for 31 *Bifidobacterium* stains isolated from infant feces.

In total, 186 isolates were isolated from 15 feces samples of 0–6-months infants. Of these, 31 strains can only be grown under anaerobic conditions at 37°C. A phylogenetic tree was constructed according to the results of 16S rRNA sequences. The results demonstrated that all isolates belonged to *Bifidobacterium*, and 24 strains among them belonged to *Bifidobacterium longum* (Figure 2).

**FIGURE 3:**
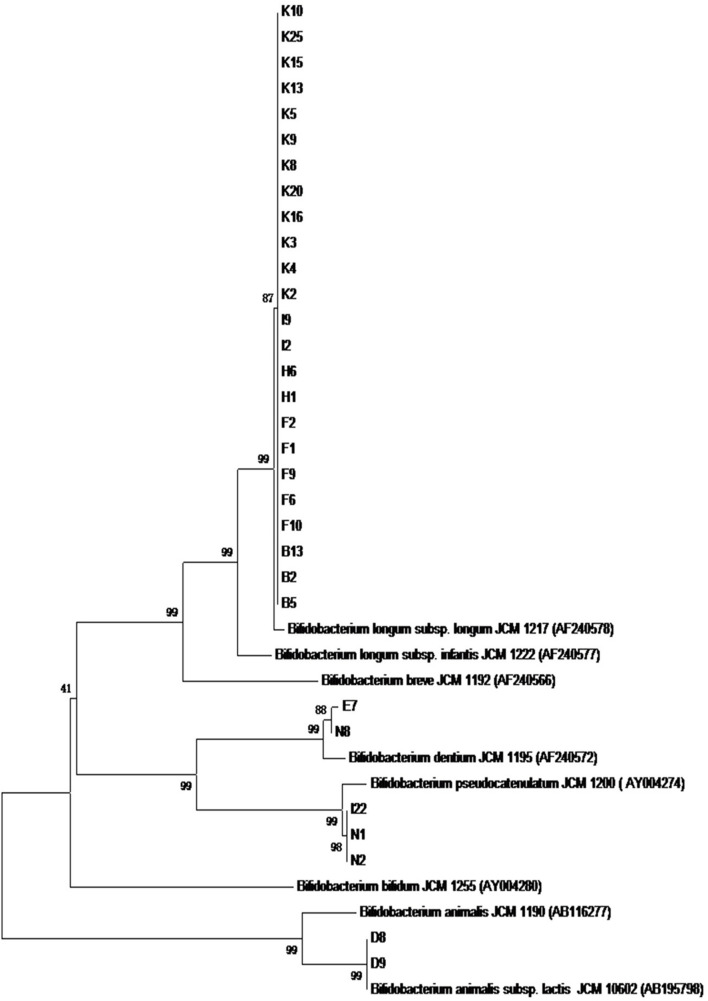
The phylogenetic tree based on the sequences of the PCR production of *hsp60* gene for 31 *Bifidobacterium* strains.

As shown in Figure 3, the phylogenetic tree was constructed according to the results of the PCR productions of *hsp60* gene sequences using the neighboring method. The results supported the findings of 16S rRNA sequences.

### Adhesion Properties

#### Autoaggregation

**FIGURE 4:**
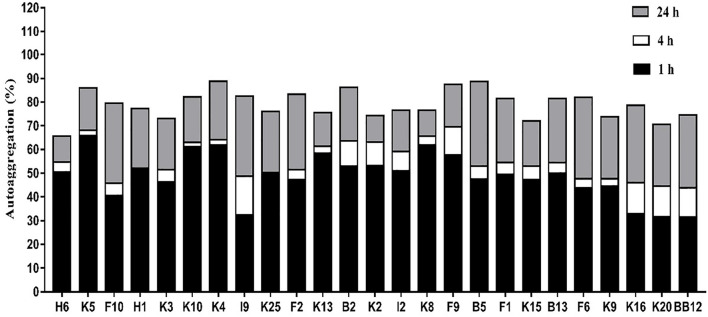
Autoaggregation of *B. longum* subsp. *longum* strains at 1h, 4h, and 24 h.

In general, all *B. longum* subsp. *longum* strains showed a high autoaggregation after incubating for 24 h (Figure 4), the autoaggregation of all *B. longum* subsp. *longum* strains were higher than that of reference strain BB-12 at 4 h. Notably, *B. longum* subsp. *longum* K5 revealed the highest autoaggregation with 65.84% at 1 h, followed by *B. longum* subsp. *longum* K10 (61.26%).

#### Cell Surface Hydrophobicity

As shown in Table 2, *B. longum* subsp. *longum* K5 presented the highest CSH level in xylene solution (69.67%), followed by K10 (64.86%) and K4 (61.45%), while *B. longum* subsp. *longum* K20 showed the lowest CSH level (21.70%). The CSH of *B. longum* subsp. *longum* isolates ranged from 21.05 to 72.18% in ethyl acetate solution. *B. longum* subsp. *longum* K5, K10, and K4 also showed significantly higher (*p* < 0.05) CSH than other strains, including the reference strain BB-12. These results indicated these 3 *B. longum* subsp. *longum* strains had good hydrophobicity ability.

**TABLE 2:** Adhesion properties of *B. longum* subsp. *longum* strains: Cell surface hydrophobicity of in xylene solution and ethyl acetate solution, and adhesion property of *B. longum* subsp. *longum* strains to HT-29 cells.

Strains	Hydrophobicity (%)		Adhesion property (%)
	
	Xylene	Ethyl acetate	
H6	38.55 ± 0.01^ef^	39.91 ± 0.01^bcd^	2.61 ± 0.62^efg^
K5	69.67 ± 0.02^a^	72.18 ± 0.02^a^	34.90 ± 2.12^a^
F10	34.09 ± 0.02^efg^	39.79 ± 0.01^bcd^	1.21 ± 0.13^fg^
H1	41.10 ± 0.02^de^	39.53 ± 0.01^bcd^	2.02 ± 0.20^fg^
K3	41.05 ± 0.02^de^	37.59 ± 0.01^bcd^	2.74 ± 0.10^efg^
K10	64.86 ± 0.02^de^	66.06 ± 0.01^a^	25.72 ± 1.64^c^
K4	61.45 ± 0.01^gh^	71.25 ± 0.02^a^	29.42 ± 1.10^b^
I9	23.43 ± 0.01^def^	21.67 ± 0.01^e^	2.75 ± 0.15^efg^
K25	40.44 ± 0.02^de^	39.44 ± 0.01^bcd^	1.80 ± 0.68^fg^
F2	41.19 ± 0.03^de^	36.52 ± 0.01^bcde^	8.23 ± 0.11^d^
K13	42.80 ± 0.02^def^	38.93 ± 0.01^bcd^	10.57 ± 1.19^d^
B2	39.83 ± 0.03^fgh^	39.73 ± 0.01^bcd^	3.04 ± 0.30^efg^
K2	28.97 ± 0.01^def^	31.09 ± 0.01^de^	0.39 ± 0.03^g^
I2	39.86 ± 0.01^ef^	41.09 ± 0.01^bcd^	1.63 ± 0.31^fg^
K8	36.51 ± 0.01^efg^	41.24 ± 0.01^bcd^	10.61 ± 3.42^d^
F9	33.55 ± 0.01^ef^	52.46 ± 0.23^b^	2.59 ± 0.27^efg^
B5	36.34 ± 0.01^efg^	34.62 ± 0.01^cde^	3.70 ± 0.18^ef^
F1	36.14 ± 0.01^ef^	29.52 ± 0.01^de^	0.30 ± 0.04^g^
B13	38.80 ± 0.01^ef^	33.53 ± 0.01^de^	0.14 ± 0.06^g^
K15	55.61 ± 0.29^bc^	39.73 ± 0.01^bcd^	5.43 ± 0.86^e^
F6	39.97 ± 0.01^def^	29.01 ± 0.01^de^	0.50 ± 0.11^g^
K9	24.53 ± 0.01^gh^	50.22 ± 0.34^bc^	0.56 ± 0.12^g^
K16	32.37 ± 0.01^efgh^	21.05 ± 0.01^e^	1.83 ± 0.21^fg^
K20	21.70 ± 0.01^h^	27.47 ± 0.02^de^	2.42 ± 0.07^fg^
BB-12	50.67 ± 0.01^cd^	30.65 ± 0.01^de^	26.33 ± 0.14^c^

*Values are conveyed as mean ± SD with independent experiments in three times. Different superscript letters in a column represent for significant differences (*p* < 0.05) in various strains.*

#### Adhesion Property to HT-29 Cells

In this study, all *B. longum* subsp. *longum* isolates showed the strain-specific property to HT-29 cells adhesion (Table 2). The adhesion property of *B. longum* subsp. *longum* strains to HT-29 cells ranged from 0.14 to 34.90%. *B. longum* subsp. *longum* K5 and K4 exhibited significantly higher (*p* < 0.05) adhesion ability than that of the reference strain BB-12 (26.33%), with the adhesion property of 34.9% and 29.42%.

#### Principal Component Analysis

**FIGURE 5:**
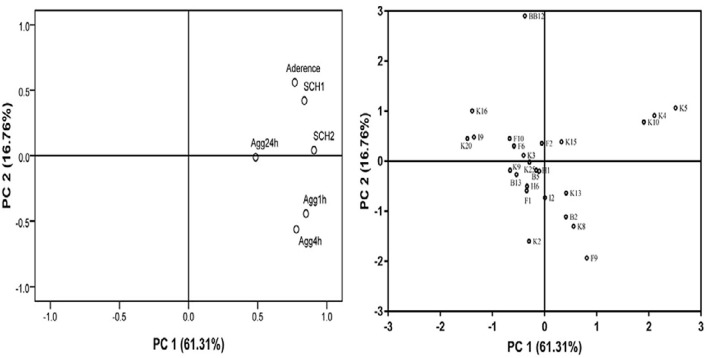
Loading plot of principal component analysis. The image in the left is loading plot represented for six independent assays of adhesion properties: autoaggregation in 1 h (Agg1 h), autoaggregation in 4 h (Agg4 h), autoaggregation in 24 h (Agg24 h), cell surface hydrophobicity level in xylene (CSH1) and ethyl acetate solution (CSH2), and adhesion property to HT-29 cells. The image in the right is score plot of the adhesion properties of *B. longum* subsp. *longum* strains.

In this study, PCA was applied for evaluating the adhesion properties of all *B. longum* subsp. *longum* strains. As shown in Figure 5, two principal components (PCs) were taken out independently. PC 1 represented 61.31% of the variance, including autoaggregation in 1, 4, and 24 h. PC 2 explained 16.76% of the variance, including CSH in ethyl-acetate and xylene and adhesion of Bifidobacteria to HT-29 cells. The total score was applied for ranking strains in this study (Supplementary Table 2). Among these strains, *B. longum* subsp. *longum* B13, F2, K4, K5, K10, K13, and K15 were extracted with a score greater than 0. The results indicated that these seven strains had good adhesion properties, and these strains were selected for further study.

### Simulation of Gastrointestinal Digestion Assay

**FIGURE 6:**
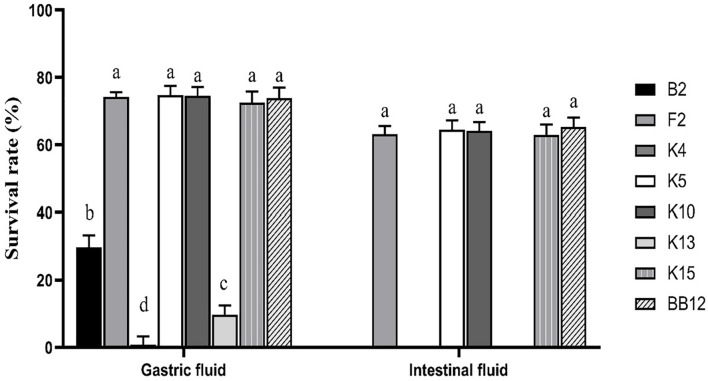
Survival rate of *B. longum* subsp. *longum* strains in the simulation of gastrointestinal digestion. *P* < 0.05 was considered to demonstrate statistically significant differences and recorded with different letters.

Figure 6 showed a survival rate of 7 *B. longum* subsp. *longum* strains treated by artificial gastrointestinal digestion *in vitro*. *B. longum* subsp. *longum* K5 showed the highest tolerance under gastric fluid with a survival rate of 74.72%, followed by *B. longum* subsp. *longum* K7 (74.53%), F2 (74.15%), and K15 (72.53%), which exhibited no significant difference (*p* > 0.05) with the reference strain BB-12 (73.91%). *B. longum* subsp. *longum* B13, K4, and K13 showed negligible survival in artificial gastrointestinal digestion conditions, excluding them from further study. After treatment of the intestinal fluid, there was no significant difference (*p* > 0.05) in survival rate among *B. longum* subsp. *longum* F2, K5, K10, K15, and BB-12 strains. Thus, *B. longum* subsp. *longum* F2, K5, K10, and K15 were selected for further study.

### Antibiotics Susceptibility

The results of antibiotic susceptibility of *B. longum* subsp. *longum* strains were accessed by using common antibiotics, as shown in Table 3. All of the *B. longum* subsp. *longum* strains were resistant to gentamicin, kanamycin, streptomycin, and ciprofloxacin. Additionally, *B. longum* subsp. *longum* K10 also exhibited resistance to clindamycin and trimethoprim (MTP). The results indicated that *B. longum* subsp. *longum* K10 were antibiotic-resistant compared to the other strains.

**TABLE 3:** Antibiotics susceptibility of the selected *B. longum* subsp. *longum* strains.

Drug class	Antibiotics	F2	K5	K15	K10
Penicillins	Ampicillin	S	S	S	S
	Penicillin G	S	S	S	S
	Amoxicillin	S	S	S	S
Glycopeptides	Gentamicin	R	R	R	R
	Kanamycin	R	R	R	R
	Streptomycin	R	R	R	R
Trimethoprim groups	Trimethoprim (MTP)	S	S	S	R
Macrolides	Roxithromycin	S	S	S	S
	Erythromycin	S	S	S	S
Tetracyclines	Tetracycline	S	S	S	S
Other	Rifampicin	S	S	S	S
	Vancomycin	S	S	S	S
	Ciprofloxacin	R	R	R	R
	Chloramphenicol	S	S	S	S
	Clindamycin	S	S	S	R

*R, resistant; S, susceptive.*

### Cell Proliferation

**FIGURE 7:**
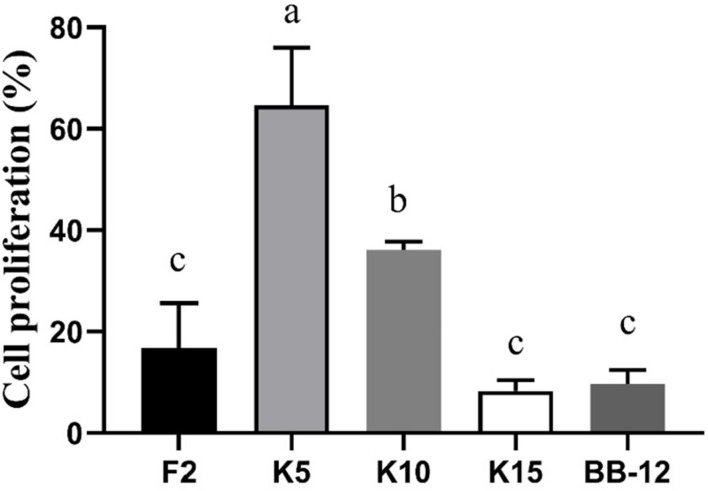
CCD 841 CoN cells proliferation of the selected *B. longum* subsp. *longum* strains. *P* < 0.05 was considered to demonstrate statistically significant differences and recorded with different letters.

As exhibited in Figure 7, the cell proliferation of CCD 841 CoN cells was evaluated by CCK-8. The cell proliferation of *B. longum* subsp. *longum* K5 (64.62%) was significantly higher (*p* < 0.05) than that of other strains, including the reference strain BB-12. There was no significant difference (*p* > 0.05) in cell proliferation among *B. longum* subsp. *longum* F2, K15, and BB-12. The results indicated that *B. longum* subsp. *longum* K5 possessed the excellent cell proliferation of CCD 841 CoN cells.

### Antioxidant Properties

#### Reducing Power

**FIGURE 8:**
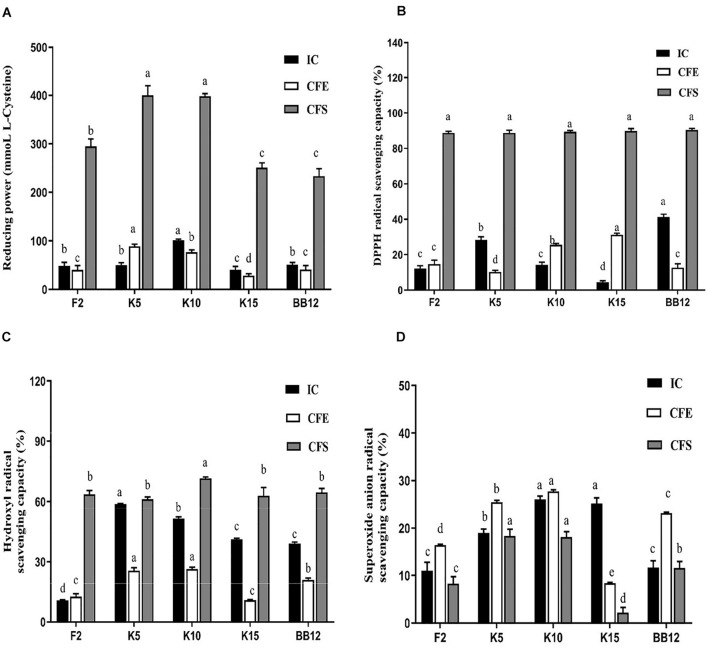
Antioxidant properties of *B. longum* subsp. *longum* strains, including reducing power **(A)**, hydroxyl radical scavenging capacity **(B)**, DPPH radical scavenging capacity **(C)**, and superoxide anion radical scavenging capacity **(D)**. IC, intact cells; CFS, cell-free supernatant; CFE, cell-free extracts. *P* < 0.05 was considered to demonstrate statistically significant differences and recorded with different letters.

All the test strains exhibited varying degrees of reducing power (Figure 8A). The IC of *B. longum* subsp. *longum* K10 showed the highest reducing power among these five strains, followed by *B. longum* subsp. *longum* K5. The IC of *B. longum* subsp. *longum* K5 exhibited no significant difference (*p* > 0.05) with BB-12. The CFE and CFS of *B. longum* subsp. *longum* K5 possessed significantly higher (*p* < 0.05) reducing power than that of reference strain BB-12. The CFS of all test strains showed higher reducing power than IC and CFE of strains.

#### DPPH Radical Scavenging Capacity

As shown in Figure 8B, the IC of BB-12 showed the highest capacity in DPPH radical scavenging, followed by *B. longum* subsp. *longum* K5. The CFE of *B. longum* subsp. *longum* K15 exhibited significantly higher (*p* < 0.05) scavenging capacity of DPPH radical than that of reference strain BB-12. The CFS of 4 *B. longum* subsp. *longum* strains displayed an excellent capacity of DPPH radical scavenging with no significant difference (*p* > 0.05). The CFS of all test strains showed higher DPPH radical scavenging capacity than IC and CFE of strains.

#### Hydroxyl Radical Scavenging Capacity

In this study, the IC and CFE of *B. longum* subsp. *longum* K5 had the highest hydroxyl radical scavenging capacity (58.76 and 25.57%), which were significantly higher (*p* < 0.05) than those of BB-12 (39.00 and 21.02%). The CFS of *B. longum* subsp. *longum* K10 expressed significantly higher (*p* < 0.05) scavenging capacity of hydroxyl radical than that of other strains (Figure 8C). The CFS of *B. longum* subsp. *longum* K5 showed no significant difference (*p* > 0.05) with BB-12. The CFS of all test strains showed higher hydroxyl radical scavenging capacity than IC and CFE of strains.

#### Superoxide Anion Radical Scavenging Capacity

As shown in Figure 8D, the superoxide anion radical scavenging capacity of IC and CFE of *B. longum* subsp. *longum* K10 (26.01 and 27.71%) and *B. longum* subsp. *longum* K5 (18.98 and 25.44%) was significantly higher than BB-12 (11.62 and 23.15%). The CFS of *B. longum* subsp. *longum* K5 and K10 showed significantly better (*p* < 0.05) superoxide anion radical scavenging capacity than other strains, including the reference strain BB-12.

## Discussion

*Bifidobacterium* is rich in breast-fed infants, especially infants aged from the first week to the sixth month (Hill C. J. et al., 2017). *Bifidobacterium* was confirmed to be beneficial to the host throughout the whole lifespan (Turroni et al., 2018). Thus, we try to isolate *Bifidobacterium* strains from infants. However, *Bifidobacterium* strains could not be isolated from all the feces samples (Tannock et al., 2016); 18% of the samples were found absent from Bifidobacteria reported by Eshaghi et al. (2017). In this study, we isolated 31 *Bifidobacterium* strains from 7 infant samples. Moreover, it was reported that the *Bifidobacterium* genus contained various species, which were rough to distinguish using 16S rRNA gene sequencing, especially *B. longum*, while the *hsp60* gene was more accurate than the 16S rRNA gene to discriminate diversified species of *Bifidobacterium* (Baffoni et al., 2013; Stenico et al., 2014). Thus, these two methods were both used in our study to identify the strains isolated from infant feces. Twenty-four strains belonged to *B. longum* subsp. *longum* in this study. We found that most of the *Bifidobacterium* strains (77.42%) belonged to *B. longum* subsp. *longum*. This finding is consistent with that of Yan et al., who isolated 93 *B. longum* subsp. *longum* strains in 30 samples contained 15 feces of adults and 15 feces of infants (Yan et al., 2017). As previously reported, *B. longum*, especially *B. longum* subsp. *longum*, have a high abundance in the gut microbiota composition of infants (Arrieta et al., 2014). Furthermore, *B. longum* subsp. *longum* verified that it could assist non-HMO metabolizing *Bifidobacterium* strains to grow moderately *via* metabolizing HMO and then benefited the development of infant gut microbiota, even the long-term health of infants (Lawson et al., 2020). Therefore, *B. longum* subsp. *longum* strains were selected for further study.

Adhesion to intestinal mucosal surfaces was considered the prerequisite for *Bifidobacterium* to exert beneficial effects on the host (Monteagudo-Mera et al., 2019). The mechanism of *Bifidobacterium* was varied from strain to strain. Thus, to evaluate the adhesion ability of *Bifidobacterium* strains, a case-by-case assay was needed. The strong autoaggregation was viewed as the first step of bacteria in the process of adhesion to cause the barrier formed by the bacteria to prevent the adhesion of pathogenic bacteria (Saito et al., 2019). In this study, most strains exhibited excellent autoaggregation, as shown in Figure 4. Moreover, cell surface hydrophobicity was considered the driver to promote the adhesion ability, and microbial adhesion to hydrocarbon was the classical method to value the level of cell surface hydrophobicity (Haddaji et al., 2015). *B. longum* subsp. *longum* B13, F2, K4, K5, K10, K13, and K15 showed higher cell surface hydrophobicity among the 24 strains in this study. Furthermore, the adhesion ability of *Bifidobacterium* strains was commonly assessed by epithelial cells such as Caco-2 and HT-29 *in vitro* to mimic adhesion to intestinal epithelial cells of humans (Tuo et al., 2013; Zuo et al., 2016; Sun et al., 2020). In this study, B13, F2, K4, K5, K10, K13, and K15 showed excellent adhesion to HT-29 cells, as exhibited in Table 2. In order to access the adhesion ability of *B. longum* subsp. *longum* strains, PCA were used to analyze the autoaggregation, cell surface hydrophobicity, and adhesion ability to HT-29 cells. As a result, *B. longum* subsp. *longum* B13, F2, K4, K5, K10, K13, and K15 possessed excellent adhesion capacity were selected for further study.

Survival in the human digestive tract (GIT) is essential for exerting probiotic function (Rabah et al., 2017; Gut et al., 2018). The reference strain BB-12 is a commercial probiotic with excellent gastric intestinal fluid tolerance, as we all know (Liu et al., 2015). Shang et al. (2020) have also assessed the ability of gastric intestinal fluid tolerance of *Bifidobacterium* strains according to the reference strain BB-12. In this study, *B. longum* subsp. *longum* F2, K5, K10, and K15 showed high tolerance of gastrointestinal digestion fluid with no significant difference with BB-12. *B. longum* subsp. *longum* B13, K4, and K13 could hardly survive in the same condition (excluded for further study).

It is essential to evaluate the antibiotics susceptibility of probiotics before being used as a dietary supplement (Borriello et al., 2003). In this study, all of the *B. longum* subsp. *longum* strains were resistant to gentamicin, kanamycin, and streptomycin only. The result was consistent with the finding of Min et al., who also evaluated the antibiotics susceptibility of *B. bifidum* BGN4 and found it was resistant to gentamicin, kanamycin, and streptomycin (Min et al., 2018). It has been reported that *Bifidobacterium* strains were generally resistant to gentamicin, kanamycin, and streptomycin and found that their resistances were intrinsic (Mtt et al., 2007). The results indicated that *B. longum* subsp. *longum* F2, K5, and K15 were susceptible to antibiotics.

The immature intestines may cause several intestinal-related diseases such as necrotizing enterocolitis and postpartum growth restriction (Hill D. R. et al., 2017; Meng et al., 2020). Thus, it is vital to promote the development of the intestine of infants. Several studies have been proved to promote the maturation of the intestines (Hill D. R. et al., 2017; Westrm et al., 2020). At the same time, the strains were not derived from the infants. This study assessed the cell proliferation of CCD 841 CoN cells used as human fetal colon epithelial cells. *B. longum* subsp. *longum* K5 possessed the excellent cell proliferation of CCD 841 CoN cells, which indicated that *B. longum* subsp. *longum* K5 could be used for drives of intestinal epithelial cell growth.

Excessive oxidative stress was harmful to humans, even infants (Hong et al., 2012; Weber et al., 2014). Excessive ROS may cause oxidative injury and cell damage (Xu et al., 2011). Thus, probiotics with the ability to reduce ROS were considered an excellent potential antioxidant. DPPH radical, reducing power (Xie et al., 2010), hydroxyl radical, and superoxide radical (Zhong et al., 2013) were used to evaluate the antioxidant capacity because of its high sensitivity and rapidity. In this study, *B. longum* subsp. *longum* K5 and K10 exhibited excellent reducing power, DPPH radical scavenging capacity, hydroxyl radical scavenging capacity, and superoxide anion radical scavenging capacity (Figure 5). It is worth noting that the CFS of test strains showed a higher antioxidant ability than that of the IC and CFE. The findings were consistent with the results of previous studies; the CFS of *B. animalis* 01 showed higher antioxidant ability than that of the IC (Shen et al., 2011). It may be caused by kinds of metabolic products, such as soluble protein (Zhang et al., 2012) and exopolysaccharides (Xu et al., 2011). It has been reported that peptides produced by lactic acid bacteria after fermentation of whey have antioxidant properties (Embiriekah et al., 2018). Liu et al. stated that exopolysaccharides possessed the antioxidant activity (Liu et al., 2011), monosaccharide composition and glycosidic linkage were proved related with the free radical scavenging activity (Torino et al., 2010).

## Conclusion

In summary, 24 *B. longum* subsp. *longum* strains were isolated from healthy infant feces. Among them, *B. longum* subsp. *longum* K5 had high adhesion ability, tolerance of artificial digestive tract, antibiotics susceptibility, intestinal cell proliferation, and antioxidant capacity. Overall, our study indicated *B. longum* subsp. *longum* K5 has excellent potential probiotic properties and antioxidant capacity and can be used as the candidate for infants’ dietary antioxidant supplements. However, most *in vivo* and clinical investigations are required before *B. longum* subsp. *longum* K5 is used for dietary supplements.

## Data Availability Statement

The original contributions presented in the study are included in the article/Supplementary Material, further inquiries can be directed to the corresponding author/s.

## Ethics Statement

The studies involving human participants were reviewed and approved by the Northeast Agricultural University Hospital Medical Science Ethics Committee. Written informed consent to participate in this study was provided by the participants’ legal guardian/next of kin.

## Author Contributions

LZ, BL, and GH conceived the study and designed the project. SW helped to collect fecal samples of infants and investigate infant information. LZ, JD, JS, and DL performed the experiment. LZ, JG, and FL analyzed the data and drafted the manuscript. All authors read and approved the final manuscript.

## Conflict of Interest

The authors declare that the research was conducted in the absence of any commercial or financial relationships that could be construed as a potential conflict of interest.

## Publisher’s Note

All claims expressed in this article are solely those of the authors and do not necessarily represent those of their affiliated organizations, or those of the publisher, the editors and the reviewers. Any product that may be evaluated in this article, or claim that may be made by its manufacturer, is not guaranteed or endorsed by the publisher.
