# Supplementary material for: Identification, Characterization, and Antioxidant Potential of Bifidobacterium longum subsp. longum Strains Isolated From Feces of Healthy Infants
Source: Front Microbiol. 2021 Nov 2;12:756519. doi: 10.3389/fmicb.2021.756519 (PMC8593421; doi:10.3389/fmicb.2021.756519)
Supplement: Supplementary file 1 [file Table_1.DOCX]

Table 1 The accession number of strains in Genbank

| Strains | Accession number |
| --- | --- |
| B2 | OK148428 |
| B13 | OK148513 |
| F1 | OK148572 |
| I2 | OK148576 |
| K3 | OK148573 |
| F6 | OK148581 |
| K2 | OK148582 |
| F9 | OK148593 |
| F10 | OK148583 |
| H1 | OK148578 |
| K20 | OK148878 |
| K15 | OK148737 |
| K10 | OK148730 |
| H6 | OK148594 |
| F12 | OK148580 |
| K5 | OK148613 |
| K9 | OK148711 |
| K8 | OK148636 |
| K4 | OK148601 |
| B5 | OK148597 |
| I9 | OK148592 |
| K13 | OK161350 |
| K16 | OK161351 |
| K25 | OK161352 |

Table 2 The total score of strains based on PCA

| Strains | Score | Strains | Score |
| --- | --- | --- | --- |
| K5 | 0.0751 | K25 | -0.0084 |
| K4 | 0.0632 | K3 | -0.0092 |
| K10 | 0.0566 | I2 | -0.0104 |
| BB12 | 0.0280 | F6 | -0.0105 |
| B13 | 0.0129 | F10 | -0.0146 |
| F2 | 0.0033 | H6 | -0.0160 |
| K13 | 0.0020 | F1 | -0.0266 |
| K15 | 0.0019 | K9 | -0.0285 |
| K8 | -0.0031 | K16 | -0.0211 |
| B2 | -0.0042 | I9 | -0.0270 |
| F9 | -0.0050 | K2 | -0.02277 |
| B5 | -0.0062 | K20 | -0.0304 |
| H1 | -0.0073 |  |  |
